# Retraction Note: Kaempferol ameliorates Cisplatin induced nephrotoxicity by modulating oxidative stress, inflammation and apoptosis via ERK and NF-κB pathways

**DOI:** 10.1186/s13568-026-02059-9

**Published:** 2026-04-25

**Authors:** Zhu Wang, Wansen Sun, Xi Sun, Ye Wang, Meilan Zhou

**Affiliations:** 1 Department of Traditional Chinese Medicine, The Second Affiliated Hospital of Xi’an Jiaotong University (Xibei Hospital), Xi’an, 710004 Shaanxi China; 2 Department of Nephropathy, Affiliated Hospital of Shaanxi University of Traditional Chinese Medicine, Xianyang, 712000 Shaanxi China; 3 Department of Nephrology, Xijing Hospital, The Fourth Military Medical University, Xi’an, 710032 Shaanxi China

**Retraction Note to: AMB Expr (2020) 10:58**

10.1186/s13568-020-00993-w

The Editor-in-Chief has retracted this article because it contains data that overlaps with data from the following article (Ye et al. 2020), also published by AMB Express. In addition, an investigation conducted after the publication determined that the two articles were under consideration by the journal at the same time. The authors did not provide an explanation for this data overlap. The Editor-in-Chief therefore no longer has confidence in the results and conclusions presented in this article.

The authors did not reply to correspondence from the Publisher about this retraction.
